# Laser Light-Based Opacitometer ‘Peira LLBO 180’: A new and validated opacitometer for use in the Bovine Corneal Opacity and Permeability (BCOP) eye irritation test method

**DOI:** 10.1016/j.mex.2020.101085

**Published:** 2020-10-02

**Authors:** An R. Van Rompay, Els Adriaens, Sandra Verstraelen

**Affiliations:** a VITO NV (Flemish Institute for Technological Research), Mol, Belgium; b Penman Consulting bvba, Brussels, Belgium; c Adriaens Consulting BVBA, Aalter, Belgium

**Keywords:** Ocular irritation, OECD Test Guideline 437, NAM, BCOP, LLBO

## Abstract

The “Peira LLBO 180” is a Laser Light-Based Opacitometer that can be used as an alternative for the standard OP-KIT device in the Bovine Corneal Opacity and Permeability (BCOP) test Organisation for Economic Co-operation and Development (OECD) Test Guideline (TG) 437 to identify chemicals inducing serious eye damage as defined by United Nations Globally Harmonized System of Classification and Labelling of Chemicals (UN GHS), *i.e.* chemicals to be classified as UN GHS Category 1 and chemicals not requiring classification for eye irritation or serious eye damage under the UN GHS classification system (No Category).

• The Peira LLBO 180 offers the advantage of analysing the complete corneal surface and is therefore able to detect more efficiently opaque spots located around the periphery of the excised corneas.

• This new device will allow not only a more accurate definition of the eye irritating potential of compounds, but also a more precise ranking of moderate to mild and non-irritating compounds.

• The value of Peira LLBO 180 is confirmed during in-house and multi-laboratory evaluation studies and is now included in the updated OECD TG 437, dated 26th of June 2020.

The results demonstrate that the presented methodology is an improved new approach methodology (NAM) for ocular irritation testing of liquids and solids.

## Graphical abstract

**Figure:**
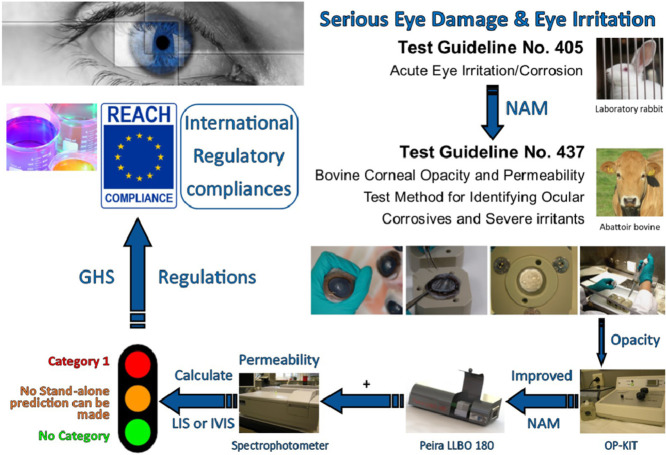

Specifications table

**Table:** 

Subject Area	Pharmacology, Toxicology and Pharmaceutical Science
More specific subject area	*In vitro* eye irritation test method
Method name	Laser Light-Based Opacitometer (Peira LLBO 180)
Name and reference of original method	1) **Research papers:** Gautheron P, Dukic M, Alix D, Sina JF [2]. Bovine corneal opacity and permeability test: an *in vitro* assay of ocular irritancy. Fundamental and Applied Toxicology. 18(3):442-9. doi:10.1016/0272-0590(92)90142-5Van Goethem, F, Hansen, E, Sysmans, M, De Smedt, A, Vanparys, P, Van Gompel, J [7]. Development of a new opacitometer for the bovine corneal opacity and permeability (BCOP) assay. Toxicol. In Vitro 24:1854-1861. doi:10.1016/j.tiv.2010.04.012**Protocols:**[4]. Protocol 124: Bovine Corneal Opacity and Permeability Assay – SOP of Microbiological Associates Ltd. Available: http://ecvam-sis.jrc.it/ICCVAM (2010). ICCVAM Recommended BCOP Test Method Protocol. In: ICCVAM Test Method Evaluation Report – Current Validation Status of In Vitro Test Methods Proposed for Identifying Eye Injury Hazard Potential of Chemicals and Products. Interagency Coordinating Committee on the Validation of Alternative Methods (ICCVAM) and the National Toxicology Program (NTP) Interagency Center for the Evaluation of Alternative Toxicological Methods (NICEATM). NIH Publication No.: 10-7553A. Available: [http://iccvam.niehs.nih.gov/methods/ocutox/MildMod-TMER.htm].
Resource availability	VITO NV: www.vito.be and Peira Scientific Instruments / Exmore: www.exmore.com

## Introduction

The Bovine Corneal Opacity and Permeability (BCOP) test method was employed by Gautheron et al. [2]. It uses isolated corneas from the eyes of freshly slaughtered cattle (calf or bovine). It is an organotypic model that provides short-term maintenance of normal physiological and biochemical function of the bovine cornea *in vitro*. In this test method, damage by the test chemical (substances and mixtures) is assessed by quantitative measurements of changes in corneal opacity and permeability with an opacitometer and a visible light spectrophotometer, respectively Table 1. Both measurements are used to calculate an OP-KIT *In Vitro* Irritation Score (IVIS) or Laser Light-Based Opacitometer (LLBO) Irritancy Score (LIS), which is used to assign an *in vitro* irritancy hazard classification category for prediction of the *in vivo* ocular irritation potential of a test chemical (OECD Test Guideline (TG) 437, 2020).

**Table 1:** Characteristics of OP-KIT and LLBO opacitometer:

Visible light-based opacitometer (OP-KIT)	Laser Light-Based Opacitometer (LLBO)
White (polychromatic) light	Laser (monochromatic) light
Two light sources of different lifetime (dual beam)	One light source (one beam)
Center-weighted reading	The whole cornea is analysed
Non-linear	Linear
The width of the light bulb can't be adjusted	The width of the light beam can be adjusted

Opacity is originally measured by an OP-KIT opacitometer [2]. The OP-KIT uses a white polychromatic light (filament bulb), it is a dual-beam opacitometer and provides a center-weighted reading of light transmission through the cornea. It measures the change in voltage when the transmission of white light passing through the cornea alters. As a consequence of the white light, this may underestimate opacity that develops as spots or heterogeneous opaque areas on the periphery of an isolated cornea and errors are also created by the use of two lamps and two photocells (control and cornea compartments). Moreover, since a decade it is difficult to commercially obtain a reliable OP-KIT opacitometer. Therefore a second opacitometer was developed, the Opacitometer 3.0 [6]. The opacity of a cornea is still measured by the diminution of light passing through the cornea. The light is now from a xenon halogen lamp, and is measured as illuminance (I = luminous flux per area, unit: lux) by a light meter (testo 545). In order to find a solution for the center-weighted reading associated with the OP-KIT opacitometer, a prototype of a Laser Light-Based Opacitometer (PLLBO) allowing better measurement of opacities was developed [7]. The prototype LLBO was further technical optimized and is now commercially available as the “Peira LLBO 180”. This LLBO uses a green diode pumped laser module as light source instead of visible light. The operation wavelength is 532 nm producing a 3.0 mW coherent, random polarized monochromatic light beam (0.81 mm) in the green portion of the visible spectrum. The LLBO offers the advantage of analyzing the complete corneal surface, and is therefore able to detect more efficiently opaque spots located around the periphery of the excised corneas [9]. The value of Peira LLBO 180 is confirmed during three in-house validation studies testing with in total 145 chemicals, and a multi-laboratory evaluation study testing the 13 proficiency chemicals from OECD TG 437, and is now included in the updated OECD TG 437, dated 26th of June 2020 [1,5,[8], [9], [10]]. The LLBO equipment is now commercially available from Exmore Benelux bvba (Beerse, Belgium).

## Procedure

This procedure is based on Protocol 124 [4] and ICCVAM-Recommended Protocol for Using the Bovine Corneal Opacity and Permeability (BCOP) Test Method ICCVAM [3]

**Fig. 1:**
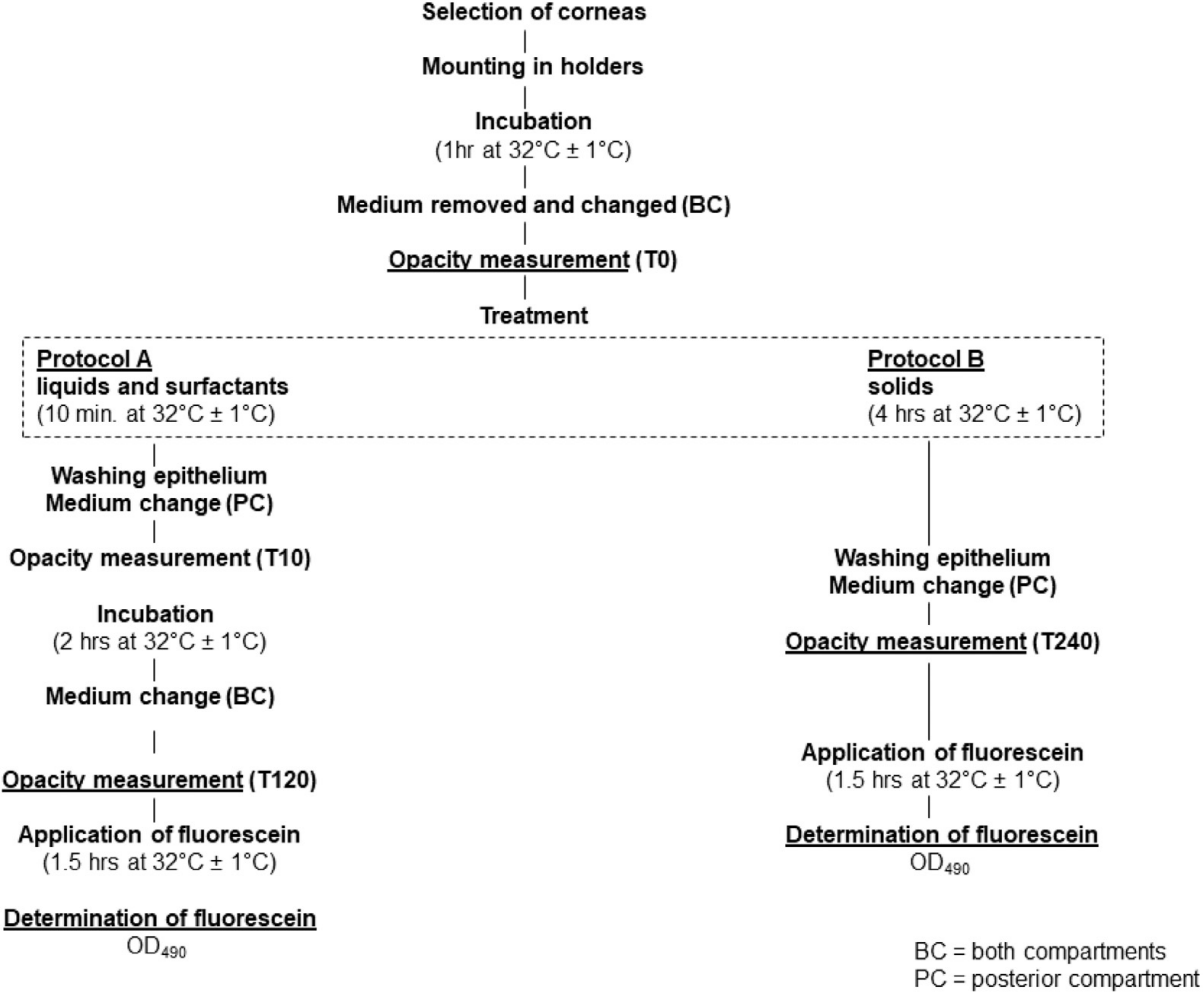
Experimental design of BCOP test method

The experimental design exists out of multiple steps as outlined in Fig. 1.

### Collection and transport conditions of the cattle eyes

A slaughterhouse veterinarian or operative will excise the eyes as soon as possible after slaughtering the cattle (calf or bovine) for consumption, the eyes are by-products. Care will be taken to avoid damaging the cornea during the enucleation procedure. Eyes will be collected in a plastic container containing 1 l of sterile 1X Hanks’ Balanced Salt Solution (HBSS, 30 eyes/l). Medium storage and transportation of eyes to the laboratory will be performed at room temperature or cooled. The eyes will be used ideally within 3 h after slaughter. The slaughterhouse and laboratory need to work according applicable regulations.

### Inspection of the eyes

**Fig. 2:**
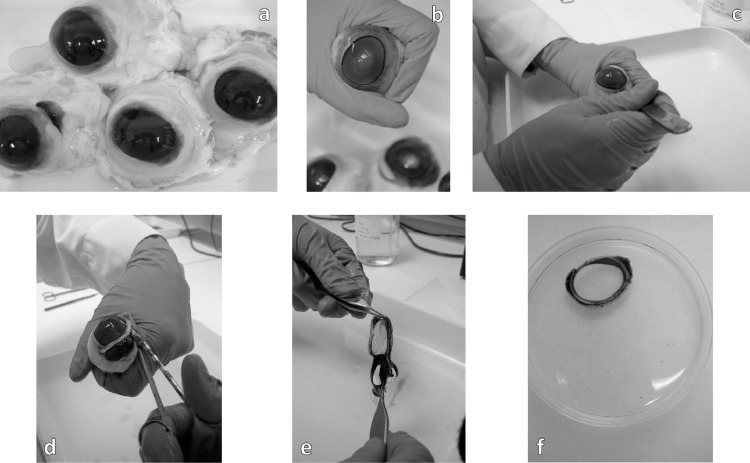
Inspection of the eyes and excising the corneas

Individual eyes will be immediately macroscopically examined (Fig. 2a+b). Those exhibiting unacceptable defects, such as opacity, scratches, pigmentation, and neovascularization will be rejected. Approximately 30% of the eyes will be discarded.

### Excising and selection of the corneas

The cornea will be carefully removed from each selected eye by making an incision (away from you) with a scalpel 2 to 3 mm outside the cornea (Fig. 2c), then by cutting around the cornea with dissection scissors (Fig. 2d), leaving a rim of sclera to facilitate handling. The iris and lens will be carefully peeled off ensuring no fragments of these tissues will be remaining on the cornea (Fig. 2e). During dissection damage to the corneal epithelium and endothelium will be avoided. Three corneas will be dissected for each exposure condition and an additional three corneas will be isolated as backup. The isolated corneas will be stored in a Petri dish containing HBSS until they will be mounted in holders (Fig. 2f).

### Mounting the corneas in the holders

**Fig. 3:**
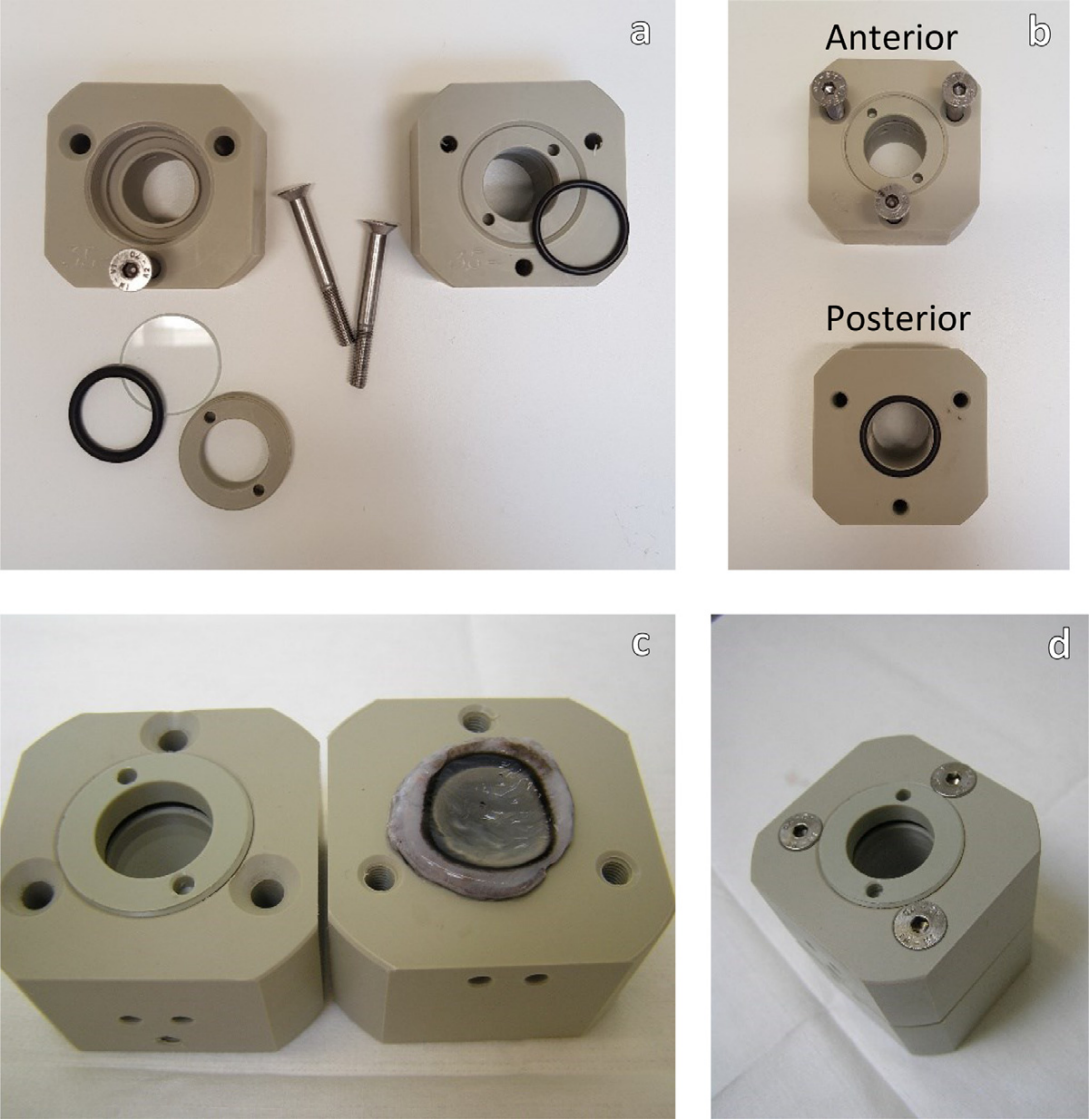
Mounting the corneas in the holder

Corneas will be handled only by the edge of the sclera to protect the epithelium and the endothelium. The wet corneas will be mounted in the polypropylene holders (one cornea per holder), by placing the endothelial side of the cornea against the O-ring of the posterior chamber. The anterior chamber will be placed over the cornea and the chambers will be joined together by tightening the chamber screws. Care should be taken not to shift the two chambers to avoid damaging the cornea (Fig. 3).

### Filling the holders with medium

**Fig. 4:**
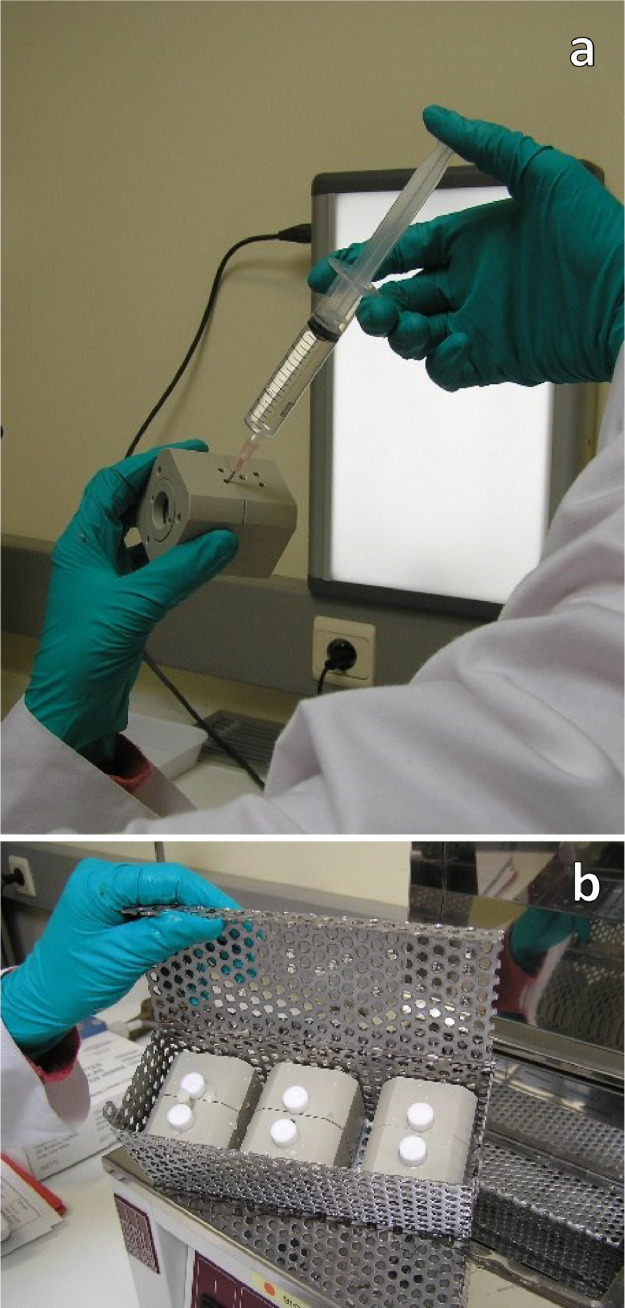
Filling the holders with medium and equilibrate the corneas

Both chambers will be filled with fresh complete pre-warmed (32 ± 1 °C) Minimum Essential Medium (MEM) (about 5 mL) by using a syringe and needle, always filling the posterior chamber first to return the cornea to its natural curvature (Fig. 4). Care should be taken when adding or removing liquid from the posterior chamber to avoid the formation of bubbles and to minimize shear forces on the corneal endothelium. Each chamber will be sealed with plugs provided with the holders and the corneas will be inspected.

### Equilibrate the corneas

The corneas will be incubated in a vertical position at 32 ± 1 °C for at least 60 min to allow the corneas to equilibrate with the medium and to achieve normal metabolic activity, to the extent possible (the approximate temperature of the corneal surface *in vivo* is 32 °C). When incubating using a water bath, 3 holders should be placed in closed water-permeable boxes to avoid floating of the holders.. At the end of the equilibration period, each cornea will be examined for defects, such as tears or wrinkling. Corneas with any observed defects will be discarded. The medium will be removed from both chambers of each holder (anterior chamber first) and replaced with fresh complete MEM. The glasses will be cleaned and dried.

### Opacity measurement (T0)

**Fig. 5:**
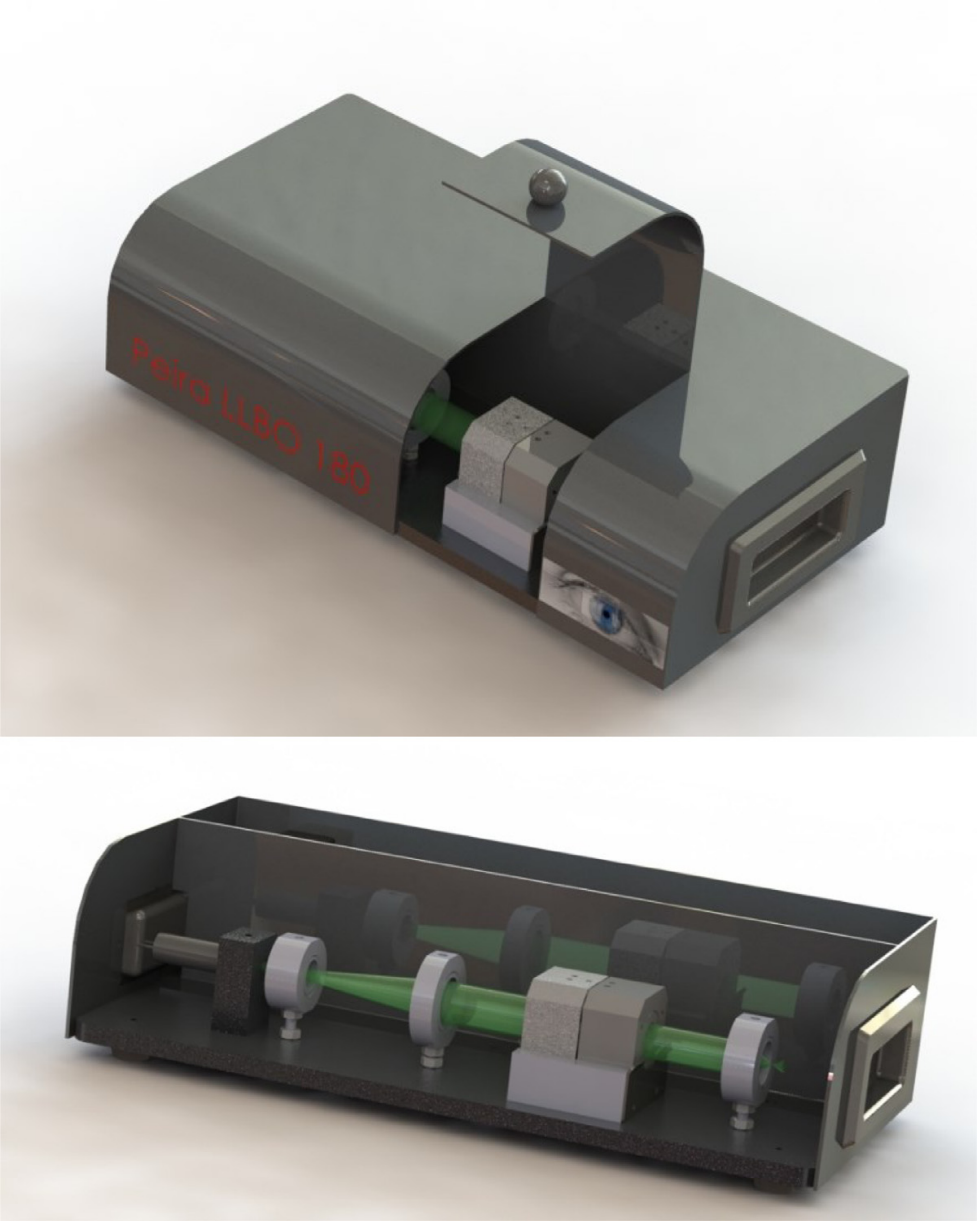
Laser Light-Based Opacitometer ‘Peira LLBO 180’

The opacitometer should provide a linear response through a range of opacity readings covering the cut-offs used for the different classifications described by the Prediction Model (*i.e.,* up to the cut-off determining corrosiveness/severe irritancy). For the LLBO equipment (Fig. 5), the opacitometer is switched on 30 min before use. It is a one-compartment/one-beam system. The unit of measurement is lux, the SI unit of illuminance. It is used in photometry as a measure of the apparent intensity of light hitting or passing through a surface, with wavelengths weighted according to the luminosity function, a standardized model of human brightness perception [1,5,9]. Start values will be set to ‘2000 ± 20 lux’ with placement of an ‘empty’ cornea holder in the measuring box, and recording lux values from illuminance meter. Next, a verification will be carried out prior to each series of measurement in order to test and guarantee that the device works faultlessly and delivers reliable measurements. The device is verified using four neutral density glass filters (OD 0.3; 0.6; 0.8 and 1.0), according to the manufacturer's specifications. After verification the device is ready to measure opacity values of the (un)treated cornea. The ‘empty’ corneal holder will be replaced by a cornea holder containing an untreated cornea, lux value (T0) will be recorded and another cornea holder will be added. After each triplicate measures, check ‘2000 lux’ reading using the ‘empty’ cornea holder and adjust if needed. Hereafter, the device is ready to measure the next three cornea holders containing untreated corneas (T0) [1,7,10].

### Test chemical preparation and their controls

All test chemical solutions (substances and mixtures) will be prepared fresh on the day of use. The test chemicals will be prepared according one of these 3 preparations.

## Non-surfactant liquid test substances

Liquid test substances are usually tested undiluted. However, if prescribed, dilutions of aqueous soluble test substances should be prepared in *e.g.* 0.9% sodium chloride. Semi-solids, creams, and waxes are typically tested as liquids.

## Non-surfactant solid test substances

Non-surfactant solid test substances are typically tested as solutions or suspensions at 20% (w/v) concentration in *e.g.* 0.9% sodium chloride. In certain circumstances and with proper scientific justification, solids may also be tested neat by direct application onto the corneal surface using the open chamber method.

## Surfactants

Neat surfactant substances are tested at a concentration of 10% (w/v, v/v) dilution or suspension in *e.g.* 0.9% sodium chloride. Appropriate justification should be provided for alternative dilution concentrations. Mixtures (*i.e.* containing one or more surfactants at a final concentration > 5%) containing surfactants may be tested undiluted or diluted to an appropriate concentration depending on the relevant exposure scenario *in vivo*. Appropriate justification should be provided for the concentration tested.

Concurrent negative or solvent controls and positive controls are included in each set of experiments.

## Negative control

A concurrent negative control, *i.e.* 0.9% sodium chloride, is included in the BCOP test so that non-specific changes in the test system can be detected and to provide a baseline for the assay endpoints. It also ensures that the assay conditions do not inappropriately result in an irritant response.

## Solvent control

When testing a diluted liquid, surfactant, or solid, a concurrent solvent control group is included in the BCOP test so that non-specific changes in the test system can be detected and to provide a baseline for the assay endpoints. Only a solvent that has been demonstrated to have no adverse effects on the test system can be used (*e.g.* 0,9% sodium chloride).

## Positive control

A known ocular irritant is included as a concurrent positive control in each experiment to verify that an appropriate response is induced. 100% ethanol (e.g. Sigma Aldrich 32221-M) or 100% N,N-dimethylformamide (≥ 99.8 % purity, *e.g.* Sigma Aldrich 319937) is typically used as a positive control for liquids and surfactants in the BCOP. The most used positive control for solid test substances is 20% (w/v) imidazole prepared in saline.

### Treatment

Three corneas will be treated with each test chemical. In addition, three corneas per assay will be treated with the concurrent positive control, and three corneas per assay will be treated with the concurrent negative control (*i.e.* 0.9% sodium chloride) or solvent/vehicle control. Each chamber will be labelled with the appropriate control or test chemical identification. Just prior to treatment, the medium will be removed from the anterior chamber through the dosing holes using an appropriate aspiration technique (*e.g.* Pasteur pipette attached to a vacuum pump).

Different treatment methods will be used depending on the physical nature and chemical characteristics (liquid or surfactant versus non-surfactant solid) of the test chemical (Fig. 1).

#### Method for non-viscous to slightly viscous liquid test item

○ Add 0.75 mL of the control or test chemical to the anterior chamber through the dosing holes using a micropipettor. The dosing holes are then resealed with the chamber plugs.
○ Rotate the holders such that the corneas are in a horizontal position. The holders should be gently tilted back and forth to ensure a uniform application of the control or test substance over the entire cornea.
○ Incubate the corneas in a horizontal position at 32 ± 1 °C for **10** **±** **1** **min**. If other exposures times are used, justification must be provided.
○ Remove the control or test chemical from the anterior chamber through the dosing holes and rinse (*e.g.* with a syringe (and needle)) the epithelium at least three times with approximately 2–3 mL of fresh complete MEM. Perform one last rinse of the epithelium using fresh complete MEM. If it is not possible to remove all visible signs of the test chemical, document the observation. Refill the anterior chamber with fresh complete MEM. Refresh the medium in the posterior chamber.
○ Observe each cornea visually and, if applicable, record pertinent observations (*e.g.* dissimilar opacity patterns, tissue peeling or residual test article).
○ Incubate the holders in a vertical (anterior chamber facing forward) position at 32 ± 1 °C for **120** **±** **10** **min**. If other post-exposure incubation times are used, justification should be provided.
○ Replace the medium in the anterior and posterior chamber with fresh complete MEM.

#### Method for semi-viscous and viscous liquid test chemicals and surfactant preparations

○ Remove the window-locking ring and glass window from all appropriate anterior chambers and place the corneas into a horizontal position (anterior chamber facing up).
○ Add test chemical to each chamber successively at a constant rate between each chamber. Apply approximately 0.75 mL of the control or test chemical (or enough test chemical to completely cover the cornea) directly to the epithelial surface of the cornea using a micropipettor or other appropriate device, such as a spatula. Maintain the corneas in a horizontal position (anterior chamber up).
○ If necessary, to aid in filling the pipette with test chemicals that are viscous, the test article may first be transferred to a syringe. Insert the pipette tip of the positive displacement pipette into the dispensing tip of the syringe, so that the test chemical can be loaded into the displacement tip under pressure. Simultaneously, depress the syringe plunger as the pipette piston is drawn upwards. If air bubbles appear in the pipette tip, the test chemical should be expelled and the process repeated until the tip is filled without air bubbles. This method should be used for any substances that cannot be easily drawn into the pipette (*e.g.* gels, toothpastes, and face creams).
○ If necessary, immediately upon dosing, slightly tilt the holders to achieve a uniform application of the test article over the entire cornea.
○ After all the chambers are dosed, replace the glass windows and window-locking rings.
○ Incubate the corneas in a horizontal position at 32 ± 1 °C for **10** **±** **1** **min**. If other exposure incubation times are used, justification should be provided.
○ Prior to the end of the exposure period, remove the window-locking ring and glass window from each appropriate chamber.
○ At the completion of the exposure period, successively rinse each cornea in the exposure group according to the intervals that they were dosed. Using a syringe, add fresh complete MEM to the inside wall of the anterior chamber creating a “whirlpool or vortex effect”, which causes the test article to be rinsed off the cornea. Take special care not to spray the medium directly onto the cornea. Residual test article that cannot be removed from the cornea by the “whirlpool method” is removed by placing a layer of medium over the cornea (added to the inside wall of the chamber). Spray a gentle stream of medium through the medium layer, directing it towards the residual test item. If after several tries the test article cannot be removed, document this, and proceed to the next step.
○ Once each cornea is completely rinsed of test chemical, replace the glass window and window-locking ring. Continue rinsing as stated previously for the “closed chamber method”.
○ Observe each cornea visually and, if applicable, record pertinent observations (*e.g.* dissimilar opacity patterns, tissue peeling or residual test article).
○ Incubate the corneas in a vertical (anterior chamber facing forward) position at 32 ± 1 °C for **120** **±** **10** **min**. If other post-exposure incubation times are used, justification should be provided.
○ Replace the medium in the anterior and posterior chamber with fresh complete MEM.

#### Solid and liquid surfactant test chemical

Surfactant test chemicals are administered following one of the previously described procedures, with one exception. Neat surfactant test chemicals are tested on the cornea as a 10 % solution or suspension prepared in an appropriate solvent/vehicle (*e.g.* 0.9% sodium chloride or sterile deionized water).

#### Solid non-surfactant test chemical

Solid non-surfactant test chemicals are administered following one of the previously described procedures, with a few exceptions, which are noted below:

○ Solid test chemicals are tested on the cornea as a 20% (w/v) solution or suspension prepared in an appropriate solvent/vehicle (*e.g.* 0.9% sodium chloride or sterile deionised water). In certain circumstances and with proper scientific justification, solids may also be tested neat by direct application onto the corneal surface using the method for semi viscous and viscous test substances.
○ Solid test chemicals are incubated at 32 ± 1 °C for **240** **±** **10** **min**.

There is no post-treatment incubation period. Thus, immediately following the rinsing process, both chambers are refilled (posterior chamber first) with fresh complete MEM.

### Opacity measurement (T120 or T240)

A post-incubation opacity reading (T120 or T240) will be recorded for each cornea, which will be used to calculate the final corneal opacity value. Each cornea will be observed visually and pertinent observations will be recorded. Special attention will be taken to observe dissimilar opacity patterns, tissue peeling or residual test substance, etc.

### Permeability measurement

The spectrophotometer will be adjusted to read at OD490. Prior to reading samples from the BCOP assay, two quality control samples of sodium fluorescein solution will be prepared and measured (see below) to ensure the sodium fluorescein calibration curve conducted for the spectrophotometer is still acceptable.

• Calibration curve for sodium fluorescein

A calibration curve for the spectrophotometer must be performed to determine the linear range and thus determine the upper limit of absorbance. Recurrent performance of the calibration curve is recommended to perform.

A first stock solution (stock 100X) will be made by dissolving 0.12 g sodium fluorescein in 100 ml Dulbecco's Phosphate Buffered Saline with Ca++ and Mg++ (DPBS). A series of dilutions will be prepared (1:1) in MEM. The OD determination is performed at 490 nm. The curve shows a linear profile between 0 and 12 µg/ml. If the result of a measurement of a sample is not in the range of the curve, a dilution will be made for a new evaluation of the sample.

• Quality control of sodium fluorescein stock

Prepare following solutions for quality control of sodium fluorescein stock

○ Liquid: sodium fluorescein (1.00 g) is dissolved in DPBS (250 ml) to make a stock solution. A dilution (1/400) in MEM is made in two steps:
Step 1: 950 µl MEM + 50 µl sodium fluorescein stock
Step 2: 500 µl of step 1 + 9500 µl MEM
○ Solid: sodium fluorescein (1.25 g) is dissolved in DPBS (250 ml) to make a stock solution. A dilution (1/500) in MEM is made in two steps:
Step 1: 950 µl EMEM + 50 µl sodium fluorescein stock
Step 2: 400 µl of step 1 + 9600 µl MEM

After zero adjustment of the spectrophotometer (2 cuvettes filled with 3000 µl MEM), optical density (OD) is determined of the step 2 solution against MEM at 490 nm. The mean OD value, calculated from the 2 measurements, must give the correct concentration on the calibration curve. If this quality criteria is fulfilled, the prepared stock solution may be divided in aliquots (falcon tubes, 15 ml) and stored in the freezer (max. 3 months).

• Application of sodium fluorescein to corneas

Following the final opacity measurement, permeability of the cornea to sodium fluorescein will be evaluated. The sodium fluorescein solution will be applied to the cornea by one of two methods, depending on the nature of the test chemical:

○ Liquid and surfactant test substances: Remove completely the medium from the anterior chamber. Add exact 1 ml of a 4 mg/ml sodium fluorescein solution to the anterior chamber using a micropipettor. Reseal the dosing holes in the top of both chambers with the chamber plugs.
○ Solid non-surfactant test substances: Remove completely the medium from the anterior chamber. Add 1 ml of a 5 mg/ml sodium fluorescein solution to the anterior chamber using a micropipettor. Reseal the dosing holes in the top of both chambers with the chamber plugs.

## Permeability determinations

○ The medium is removed from both chambers and fresh medium added to the posterior chamber without causing bubbles in the chamber.
○ After adding the sodium fluorescein to the anterior chamber and sealing the chambers, rotate the corneas into a horizontal position with the anterior chamber facing up. Tilt the holders slightly, if necessary, to achieve a uniform application of the sodium fluorescein over the entire cornea. Incubate the holders in a horizontal position for 90 ± 5 min at 32 ± 1 °C.
○ After the 90-minute incubation period, remove the medium with a syringe or Pasteur pipette in the posterior chamber of each holder and place into sample tubes pre-labelled according to holder number. It is important to remove most of the medium from the posterior chamber and mix it in the tube so that a representative sample can be obtained for the OD490 determination.
○ If the average of the quality control samples falls within the accepted range of the calibration curve, then proceed to read samples from the BCOP assay. Transfer an aliquot of the mixed medium from the posterior chamber of the BCOP holder into a cuvette, then take an absorbance reading using the spectrophotometer. Any solutions giving an OD490 beyond the linear range of the spectrophotometer must be diluted in complete MEM, and another reading taken, repeating these steps until the OD490 is within the linear range of the spectrophotometer. Repeat these procedures for each sample from the BCOP assay until all samples have been read and results recorded.
○ Alternatively, a 96-well microtiter plate reader may be used provided that (i) the linear range of the plate reader for determining fluorescein OD490 values can be established, and (ii) the correct volume of fluorescein samples are used in the 96-well plate to result in OD490 values equivalent to the standard 1 cm path length (*i.e.* a completely full well, 360 µl).
○ At the end the corneas and other tissues are discarded in accordance with local regulations.

### Data evaluation

Results from the two test method endpoints, opacity and permeability, should be combined in an empirically derived formula that generates an LLBO Irritancy Score (LIS) for each test chemical.

○ Opacity
○ Calculate the change in opacity for each individual cornea (including the negative control) by subtracting the initial opacity reading from the final post-treatment opacity reading. Then calculate the average change in opacity for the negative control corneas.
○ Calculate a corrected opacity value for each treated cornea, positive control, and solvent/vehicle control (if applicable) by subtracting the average change in opacity of the negative control corneas from the change in opacity of each treated, positive control, or solvent/vehicle control cornea.
○ Calculate the mean opacity value of each treatment group by averaging the corrected opacity values of the treated corneas for each treatment group.

## Permeability

○ Calculate the corrected OD_490_ value of each treated, positive control, or solvent/vehicle control cornea by subtracting the average value of the negative control corneas from the original OD_490_ value for each cornea. Final corrected OD_490_ = raw OD_490_ - mean blank corrected negative control OD_490_.
○ Calculate the mean OD_490_ value for each treatment group by averaging the final corrected OD_490_ values of the treated corneas for a particular treatment group.

## LLBO Irritancy Score (LIS)

The unit for the opacity measurement is lux, the SI unit of illuminance, and the opacity is defined as mean lux/7, in order to transform the obtained illuminance values in the range of the standard OP-KIT prediction model. Use the mean opacity and mean permeability values (OD_490_) for each treatment group to calculate an *in vitro* score for each treatment group:

*LIS* = mean opacity value (read-out of LLBO in lux/7) + (15 x mean permeability value).

### Study acceptance criteria

A test is acceptable if the positive control induces an LIS score in the severe range. The negative or solvent/vehicle control responses should result in *LIS value* ≤ 30.

A single testing run composed of at least three corneas should be sufficient for a test chemical when the resulting classification is unequivocal. However, in cases of borderline results the instructions from OECD TG 437 [5] should be followed.

### Classification

**Fig. 6:**
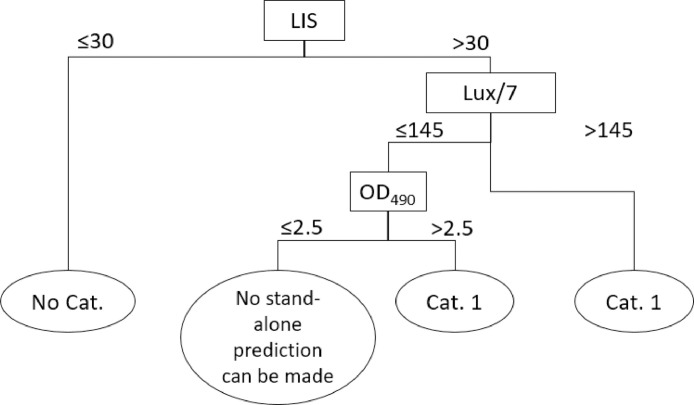
Prediction model for BCOP test method with Laser Light-Based Opacitometer “Peira LLBO 180”

The prediction model for identifying test chemicals as inducing serious eye damage (UN GHS Category 1) and test chemicals not requiring classification for eye irritation or serious eye damage (UN GHS No Category) are given in Table 2 and Fig. 6.

**Table 2:** Classification for eye irritation with Laser Light-Based Opacitometer “Peira LLBO 180”

LLBO*	UN GHS
LIS ≤ 30	No Category
LIS > 30 and lux/7 ≤ 145 and OD_490_ ≤ 2.5	No stand-alone prediction can be made
• LIS > 30 and lux/7 ≤ 145 and OD_490_ > 2.5 or • LIS > 30 and lux/7 > 145	Category 1

## Method validation

The reproducibility of the BCOP LLBO protocol for liquids and solids and its predictive capacity is demonstrated [1,5]. Briefly, 145 chemicals were simultaneously tested with the 2 opacitometers LLBO and OP-KIT. The LLBO was found to have a higher sensitivity (identify Category 1) compared to OP-KIT, the sensitivity (based on 56 Category 1 chemicals) increased from 65.2% (OP-KIT) to 75.9% (LLBO), with a comparable false positive rate of 18.5% and 20.8%, respectively. When used to identify chemicals not requiring classification (No Category) the OP-KIT and LLBO had a false negative rate (*N* = 104) of 7.2% and 6.2% and a false positive rate (*N* = 41) of 42.7% and 45.1%, respectively. Furthermore, the technical proficiency of the BCOP LLBO test method was assessed in 3 laboratories. Technical competence in performing the BCOP LLBO Performance of the 13 proficiency chemicals is demonstrated [8].

## Conclusion

The ultimate goal of this study was to improve the performance of the OP-KIT opacitometer. For this purpose we constructed the Laser Light-Based Opacitometer ‘Peira LLBO 180’. The OP-KIT and LLBO devices are interchangeable at no cost to data quality and reliability. LLBO improves the performance of BCOP test, at the level it can be integrated within a testing strategy to perform an eye irritation risk assessment. This efficient transferable and reproducible LLBO opacitometer is a promising NAM, and is recently added in OECD TG 437 and is to be integrated within a battery of test methods (Integrated Approach on Testing and Assessment (IATA) and Defined approaches (DA)) to perform an eye irritation risk assessment.

Supplementary material *and/or* Additional information:

## Declaration of Competing Interest

The authors declare that they have no known competing financial interests or personal relationships that could have appeared to influence the work reported in this paper.

## Appendix Supplementary materials

Image, video 1
